# Galactose Oxidase from *Fusarium oxysporum* - Expression in *E. coli* and *P. pastoris* and Biochemical Characterization

**DOI:** 10.1371/journal.pone.0100116

**Published:** 2014-06-26

**Authors:** Regina Paukner, Petra Staudigl, Withu Choosri, Christoph Sygmund, Petr Halada, Dietmar Haltrich, Christian Leitner

**Affiliations:** 1 Department of Food Science and Technology, BOKU-University of Natural Resources and Life Sciences, Vienna, Austria; 2 Department of Food Technology, Ramkhamhaeng University, Bangkok, Thailand; 3 Institute of Microbiology v.v.i., Academy of Sciences of the Czech Republic, Prague, Czech Republic; Instituto de Tecnologica Química e Biológica, UNL, Portugal

## Abstract

A gene coding for galactose 6-oxidase from *Fusarium oxysporum* G12 was cloned together with its native preprosequence and a C-terminal His-tag, and successfully expressed both in *Escherichia coli* and *Pichia pastoris*. The enzyme was subsequently purified and characterized. Among all tested substrates, the highest catalytic efficiency (*k*_cat_/K_m_) was found with 1-methyl-β-D-galactopyranoside (2.2 mM^−1 ^s^−1^). The Michaelis constant (K_m_) for D-galactose was determined to be 47 mM. Optimal pH and temperature for the enzyme activity were 7.0 and 40°C, respectively, and the enzyme was thermoinactivated at temperatures above 50°C. GalOx contains a unique metalloradical complex consisting of a copper atom and a tyrosine residue covalently attached to the sulphur of a cysteine. The correct formation of this thioether bond during the heterologous expression in *E. coli* and *P. pastoris* could be unequivocally confirmed by MALDI mass spectrometry, which offers a convenient alternative to prove this Tyr-Cys crosslink, which is essential for the catalytic activity of GalOx.

## Introduction

Galactose oxidase (GalOx; D-galactose:oxygen 6-oxidoreductase, EC 1.1.3.9) is a member of the radical copper oxidase family [1] and has been classified as a member of the carbohydrate active-enzyme family AA5, subfamiliy 2 [2]. It is an extracellular monomeric enzyme [3], [4] secreted by several filamentous fungi [5]–[9], with the enzyme from *Fusarium graminearum* (formerly classified as *Dactylium dendroides*) being perhaps the best studied representative. GalOx catalyzes the oxidation of primary alcohols (e.g., the hydroxyl group at the C6 position in D-galactose) to aldehydes, accompanied by the reduction of molecular oxygen to hydrogen peroxide [10] according to the following reaction scheme:
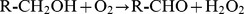



GalOx is characterized by a broad substrate tolerance, yet strict stereospecificity, for various alcohol substrates [11].

Typically, wild-type fungal GalOx is produced as a prepro form carrying a N-terminal signal sequence, which is removed upon secretion, yielding the immature proform. The prosequence in this form was suggested to function as an intramolecular chaperone supporting copper binding and cofactor formation [4]. Removal of the prosequence by a tryptic-like cleavage then generates the correct N-terminus of the mature protein [12]. GalOx is unusual among metalloenzymes in catalyzing a two-electron redox chemistry at a mononuclear metal ion active site [13], with a tyrosine radical serving as the second redox cofactor. In *F. graminearum* GalOx, Tyr272, one of the copper ligands, is covalently linked at C_ε_ to the sulphur atom of Cys228. This bond seems to have partial double-bond character with C_β_ of Cys228 lying in the same plane as the ring of Tyr272 [3]. The formation of the Tyr^•^-Cys redox cofactor in GalOx is a self-processing reaction requiring only the apoprotein, copper, and dioxygen; no other proteins or enzymes are required for the processing and assembly of the catalytically active enzyme [14]. Furthermore, the Tyr-Cys cross-link has been shown to form spontaneously on exposure to excess Cu(II) even in the absence of oxygen [15]. GalOx exists in three different oxidative states. The oxidized (Cu^II^, Tyr^•^) and the reduced form (Cu^I^, Tyr) are involved in the catalytic cycle. The semi-reduced form (Cu^II^, Tyr) is catalytically inactive but can be oxidized to the catalytically active oxidized form [16]. The copper in the active site is coordinated by four amino acid side chains: two tyrosines (Tyr272 and Tyr495) and two histidines (His496 and His581) [17].

Wild type GalOx of *F. graminearum* has been cloned and expressed in *E. coli* with low yields [18]–[20]. Expression has been increased by using directed evolution and site directed mutagenesis [21]–[23]. Higher yields were obtained by expression of the wild type enzyme in *P. pastoris* [15], [24]–[27] and *A. nidulans* [28].

In this study, we report the successful heterologous expression of a novel, not-yet-characterized GalOx from *F. oxysporum* both in *E. coli* and *P. pastoris*. We investigated the biochemical properties of the enzyme, and for the first time confirmed the formation of the unique thioether bond in both expression systems directly using mass spectrometry.

## Materials and Methods

### Materials

All chemicals were reagent-grade or better and purchased from Sigma-Aldrich (Steinheim, Germany) unless otherwise stated. 2,2′-Azino-bis(3-ethylbenzthiazoline-6-sulfonic acid) (ABTS) was obtained from Amresco (Solon, OH, USA). Restriction enzymes, ligase and standards for Agarose gel electrophoresis (GeneRuler DNA Ladder Mix) were obtained from Fermentas (Vilnius, Lithuania) while the Phusion polymerase was from New England BioLabs (Ipswich, UK). SDS-PAGE protein standard (Precision Plus Protein prestained standard) was from BioRad (Herts, UK). The cloning vector pJET 1.2 was purchased from Fermentas and the expression vector pET21a from Novagen (Madison, WI, USA). The expression vectors pPICZB and pPICZαA, *E. coli* strain BL21 (DE3) and *P. pastoris* strain X-33 were purchased from Invitrogen (Carlsbad, CA, USA). *E. coli* NEB 5-alpha was from New England BioLabs. The HisPrep FF 16/10 column was from GE Healthcare Bioscience AB (Uppsala, Sweden). *F. oxysporum* strain G12 was kindly provided by Gerhard Adam (Department of Applied Genetics and Cell Biology, BOKU Vienna, Austria).

### Isolation and cloning of the GalOx gene

*Fusarium oxysporum* strain G12 was cultivated in shaken flasks for 2 days at 25°C and 110 rpm in Sabouraud medium (5 g L^−1^ peptone from casein, 5 g L^−1^ peptone from meat, 10 g L^−1^ glucose, 10 g L^−1^ maltose, 5 g L^−1^ yeast extract). Fungal mycelium was collected by centrifugation (4°C, 15 min at 5,000×g) and washed. Subsequently, genomic DNA was isolated from 100 mg of frozen mycelia ground in liquid nitrogen using the Wizard SV Genomic DNA Purification System (Promega; Madison, WI, USA). The GalOx gene including its prepro sequence was amplified by PCR using primers based on the published genome of *F. oxysporum* [29] (FOXG-09956.29): 5′-ATGAAGCCCCTTTGGACACTTGC-3′ and 5′-CTACTGAGTAACGAGAAGAGTACTCGC-3′. The resulting PCR product of approximately 2 kb was purified from the agarose gel using the Wizard SV Gel & PCR CleanUp System (Promega) and ligated into the pJET 1.2 vector using the CloneJET PCR Cloning Kit (Fermentas).

For expression in *E. coli Nde*I and *Sal*I restriction sites were introduced by PCR using the following Primers (restriction sites underlined): 5′-CAGTGCATATGAAGCCCCTTTGGACAC-3′ and 5′-CGAAGTCGACCTGAGTAACGAGAAGAG-3′. After digestion with *Nde*I and *Sal*I the PCR product was ligated into the multiple cloning site of the expression vector pET21a in frame with the C-terminal His_6_-tag and transformed into *E. coli* BL21 (DE3). To confirm the correct sequence, the recombinant plasmid was analyzed by restriction digestion and sequenced by VBC Biotech (Vienna, Austria). Two different vectors were constructed for expression in *P. pastoris*. The first vector contained the full-length GalOx cDNA including the native prepro signal. In the second vector this sequence was replaced by the α-factor of *S. cerevisiae*, which serves as secretion signal for the mature protein. For the first construct the full-length GalOx cDNA was cloned into the expression vector pPICZB under control of the methanol-inducible AOX promoter and in frame with a C-terminal His_6_-tag. To this end, the restriction sites *EcoR*I and *Not*I were introduced by PCR (Primer: 5′-TCGCAGAATTCATGAAGCCCCTTTGGACACTTG-3′; 5′-GTCATGCGGCCGCCTGAGTAACGAGAAGA-3′), and after digestion the PCR product was ligated into the vector using the Quick Ligation Kit (New England BioLabs). The ligation product was transformed into the electrocompetent *E. coli* strain NEB 5-alpha, and positive clones were selected on Low Salt LB agar plates containing 25 µg mL^−1^ Zeocin. Plasmid DNA was isolated using the Pure Yield Plasmid Miniprep System (Promega) and linearized with *Sac*I within the 5′ AOX1 region to promote integration. The restriction enzyme was heat inactivated and the plasmid purified with the Wizard SV Gel and PCR Clean-Up System (Promega). Electrocompetent *P. pastoris* X-33 cells were prepared and transformed with the linearized plasmid according to the operating instructions and applications guide of the MicroPulser electroporation apparatus (BioRad). Transformants were selected for growth on YPD plates containing 100 µg mL^−1^ Zeocin. In the second construct the prepro sequence of the mature GalOx cDNA was replaced with the α-mating factor of *S. cerevisiae*. To produce the truncated cDNA a different forward primer was used for PCR (5′-TCGCAGAATTCATGGCCTCAGCTCCCATCG-3′) and the resulting PCR product was ligated into the pPICZαA vector as described above.

### Heterologous expression

Cultivation of *E. coli* BL21 (DE3) for production of the recombinant enzyme was performed in double concentrated LB medium (20 g L^−1^ peptone from casein, 10 g L^−1^ yeast extract, 10 g L^−1^ NaCl, and 5 µM CuSO_4_) containing 50 mg L^−1^ ampicillin to maintain the expression plasmid. *E. coli* was grown in baffled shaking flasks at 37°C until an OD_600_ of 0.4–0.6 was reached. Recombinant protein production was induced by the addition of 5% lactose and cultivation was continued at 25°C for 16 h [30].

Production of recombinant GalOx in *P. pastoris* was also carried out in baffled shaken flasks. Precultures (20 mL) were grown at 30°C in YPD medium containing 25 µg mL^−1^ Zeocin. After 16 h the precultures were used to inoculate 1-L baffled shaken flasks containing 200 mL of BMMY medium (buffered methanol complex medium) and further incubated at 30°C. After 3 days a linear methanol feed (2% per day) was started. Samples were taken every day, clarified by centrifugation, and protein concentrations as well as GalOx activities were assayed in the supernatant.

### Enzyme purification

Recombinant GalOx was purified from both, the *E. coli* biomass and the *P. pastoris* supernatant, respectively. *E. coli* biomass was resuspended in phosphate buffer (20 mM, pH 7.0) after harvesting the cells by centrifugation (4°C, 30 min at 6,000×g). Cell disruption was performed by addition of lysozyme (1 mg mL^−1^) and incubation for 30 min at 37°C, followed by sonication, as previously described [30]. Cell debris was removed by ultracentrifugation (30 min at 30,000×g and 4°C). The clear supernatant was applied to a HisPrep FF 16/10 column previously equilibrated with phosphate buffer (50 mM, 500 mM NaCl, pH 8.0) and eluted in a linear gradient from 0–1 M imidazole in 10 column volumes. Active fractions were pooled, concentrated using an Amicon Ultra Centrifugal Filter Device with a 30-kDa cut-off membrane (Millipore; Billerica, MA, USA), and further purified by size-exclusion chromatography (HiPrep Sephacryl HR 16/60, 50 mM phosphate, 150 mM NaCl, pH 6.5). Active fractions were collected, concentrated and stored at −30°C. Recombinant GalOx from *Pichia* cultivations was purified from the culture supernatant as described above after cells had been removed by centrifugation (6,000×g; 30 min; 4°C).

### Electrophoresis

SDS-PAGE was performed as described by Laemmli [31] using a 5% stacking gel and a 10% separating gel. The system used was the PerfectBlue vertical electrophoresis apparatus (Peqlab, Erlangen, Germany). Precision Plus Protein Dual Color was used as molecular mass standard. Gels were stained with Coomassie Brilliant Blue (CBB).

### Circular dichroism spectrum

Far UV electronic circular dichroism (CD) spectra of GalOx from *F. oxysporum* were recorded using a Chirascan CD Spectrophotometer (Applied Photophysics, Leatherhead, UK) in the wavelength range of 180 to 260 nm. The instrument was flushed with nitrogen and the conditions were set as follows: path length was 1 mm, spectral bandwidth 3 nm and the scan time per point was set to 10 s. CD measurements were performed with 0.5 mg mL^−1^ GalOx in 5 mM potassium phosphate buffer (pH 7.0).

### Enzymatic in-gel digestion and MALDI mass spectrometry

Coomassie brilliant blue stained protein bands were excised from the gel, cut into small pieces and washed with 50 mM 4-ethylmorpholine acetate (pH 8.1) in 50% acetonitrile (MeCN). After complete destaining, the supernatant was removed and the gel was partly dried in a SpeedVac concentrator. The protein digestion using 100 ng of sequencing-grade trypsin (Promega, Madison, WI) was performed overnight at 37°C in a cleavage buffer containing 25 mM 4-ethylmorpholine acetate (pH 8.1) and 5% MeCN. Alternatively, the proteins were digested sequentially by trypsin (as described above) and Asp-N protease (20 ng, 6 h; Roche, Mannheim, Germany) as well as using the sequence Asp-N and trypsin, always in the same cleavage buffer. The digestions were stopped by addition of 5 µL of 5% trifluoroacetic acid in MeCN. An aliquot of the peptide mixture (0.5 µL) was deposited on the MALDI target and allowed to air-dry at room temperature. After complete evaporation, 0.5 µL of the matrix solution (α-cyano-4-hydroxycinnamic acid in 50% MeCN/0.1% TFA; 5 mg/ml) was added.

MALDI mass spectra were measured on an Ultraflex III MALDI-TOF/TOF instrument (Bruker Daltonics, Bremen, Germany) equipped with LIFT technology for MS/MS analysis. MALDI mass spectra were also obtained on a high accuracy APEX-Qe FT-ICR instrument equipped with a 9.4 T superconducting magnet and a Combi ESI/MALDI ion source (Bruker Daltonics, Billerica, MA). The spectra were acquired in the mass range of 700–5000 Da and calibrated internally using the monoisotopic [M + H]^+^ ions of trypsin autoproteolytic fragments (842.5 and 2211.1 Da).

### Enzyme activity assay

Prior to activity measurements GalOx was activated by incubation with 1 mM CuSO_4_ for 30 min and 25°C (Thermomixer comfort, Eppendorf, Hamburg, Germany; 800 rpm). The activity was determined with the chromogenic 2,2′-azinobis(3-ethylbenzthiazolinesulfonic acid) (ABTS) assay [32]. The standard reaction mixture with a total volume of 1 mL contained 1 µmol of ABTS in 20 mM potassium phosphate buffer (pH 7.0), 2 U of horseradish peroxidase, and 100 µmol of galactose. The reaction was started by adding 20 µL of a suitably diluted enzyme solution. The absorbance change at 420 nm was recorded at 30°C for 180 s (ε_420_ = 43.2 mM^−1 ^cm^−1^). One Unit of GalOx activity was defined as the amount of enzyme that is necessary for the oxidation of 2 µmol of ABTS per min, which equals the consumption of 1 µmol of O_2_ per min, under the conditions described above. A substantially identical activity assay was used for determination of the substrate specificity of GalOx, however, galactose was replaced by 100 µmol of the respective substrate in the reaction mixture. Protein concentrations were determined by the dye-binding method of Bradford [33] using the BioRad Protein Assay Kit with BSA as a standard. All tests were performed in duplicates.

### Steady-state kinetic measurements

Kinetic constants were determined for GalOx produced in *E. coli* at 30°C in 20 mM potassium phosphate buffer (pH 7.0) for different sugar substrates. D-galactose (1–500 mM), 1-methyl-β-D-galactopyranoside (5–200 mM), melibiose (1–250 mM), as well as raffinose, lactose, 1-methyl-α-D-pyranoside, lactulose, 2-deoxy-D-galactose and lactitol, each varied over a range of 10–250 mM, were used as substrates. All kinetic constants were calculated using non-linear least-square regression by fitting the observed data to the Michaelis-Menten equation (Sigma Plot 9, Systat; Chicago, IL, USA). Turnover numbers (*k_cat_*) were calculated using a theoretical molecular mass of 73.9 kDa.

### pH dependence of activity

A pH-activity profile was determined in the range of pH 2.5 to 10.0 using the buffer system citric acid (pH 2.5–6.0), potassium phosphate (pH 6.0–8.0) and Tris (pH 8.0–10.0), each at a concentration of 50 mM. Activity measurements were performed otherwise as described above for the standard assay.

### Temperature optimum and thermal stability

Determination of the temperature optimum of GalOx was achieved by measuring the activity with the standard assay at different temperatures in the range of 30 to 65°C. Thermal stability of GalOx was determined by incubating the protein at 30, 40, 50 and 60°C. Samples were taken at various time points, cooled on ice, and residual GalOx activity was measured using the standard ABTS assay after reactivating the enzyme by incubation with CuSO_4_. Differential scanning calorimetric (DSC) measurements were performed using a MicroCal VP-Capillary DSC system (GE Healthcare) controlled by the VP-viewer program and equipped with an active cell volume of 130 µL. To check the influence of different buffers on the stability of the protein the studies were made with 4 µM GalOx in 20 mM citric acid, phosphate, and Tris buffer each at pH 7. Samples were analyzed using a heating scan rate of 60°C h^−1^ over a temperature range of 15–100°C. Collected DSC data were corrected for baseline with the corresponding buffer. Data analysis was performed with the MicroCal Original software.

## Results and Discussion

### Isolation, cloning and expression of GalOx in *E. coli* and *P. pastoris*

The *gao* gene encoding GalOx was successfully amplified together with its signal sequence from genomic DNA of the fungus *F. oxysporum* G12. The gene consists of an open reading frame of 2043 bp encoding a polypeptide of 681 amino acids. The sequence (accession number KF601698) contains no introns and shows 99% similarity to the published amino acid sequence for GalOx from *F. oxysporum* [29] showing five changes in its sequence (A169T, P190A, S402T, R413Q, and V487I). Furthermore, the *gao* gene has 96%, 62%, and 67% similarity with the *gaoA* (BK007071), *gaoB* (BK007067) and *gaoC* (BK007074) gene from *F. oxysporum* f. sp. *lycopersici*, respectively [8]. Hence the cloned *gao* gene from *F. oxysporum* belongs to the *gaoA* cluster of GalOx genes. The similarity to the protein sequence of GalOx from *F. graminearum* [3] was 82%. No second *gao* gene was found under the conditions used.

**Figure 1:**
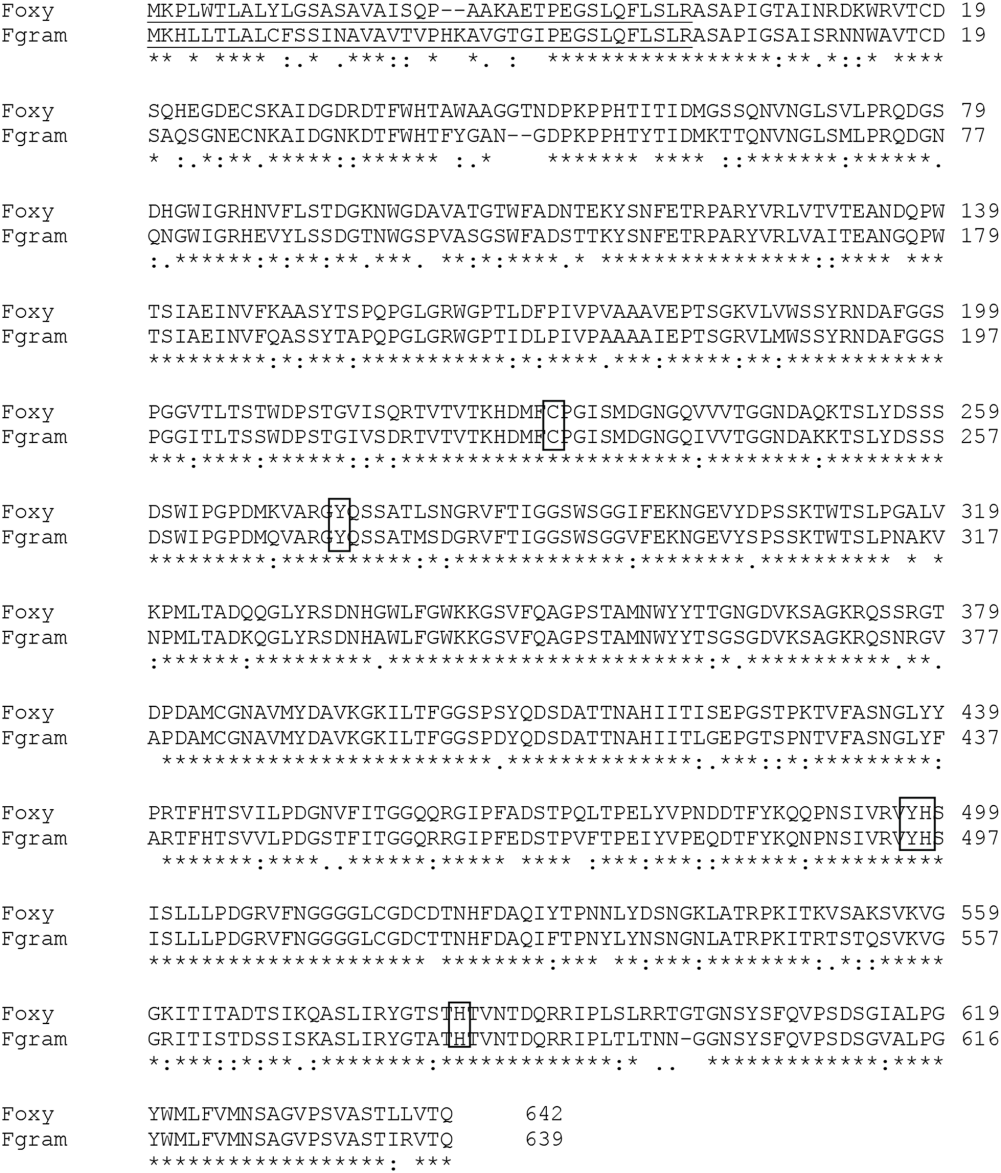
Alignment of GalOx from *F. oxysporum* and *F. graminearum*. The prepro sequence is underlined. Amino acid residues involved in copper binding are highlighted.

**Figure 2:**
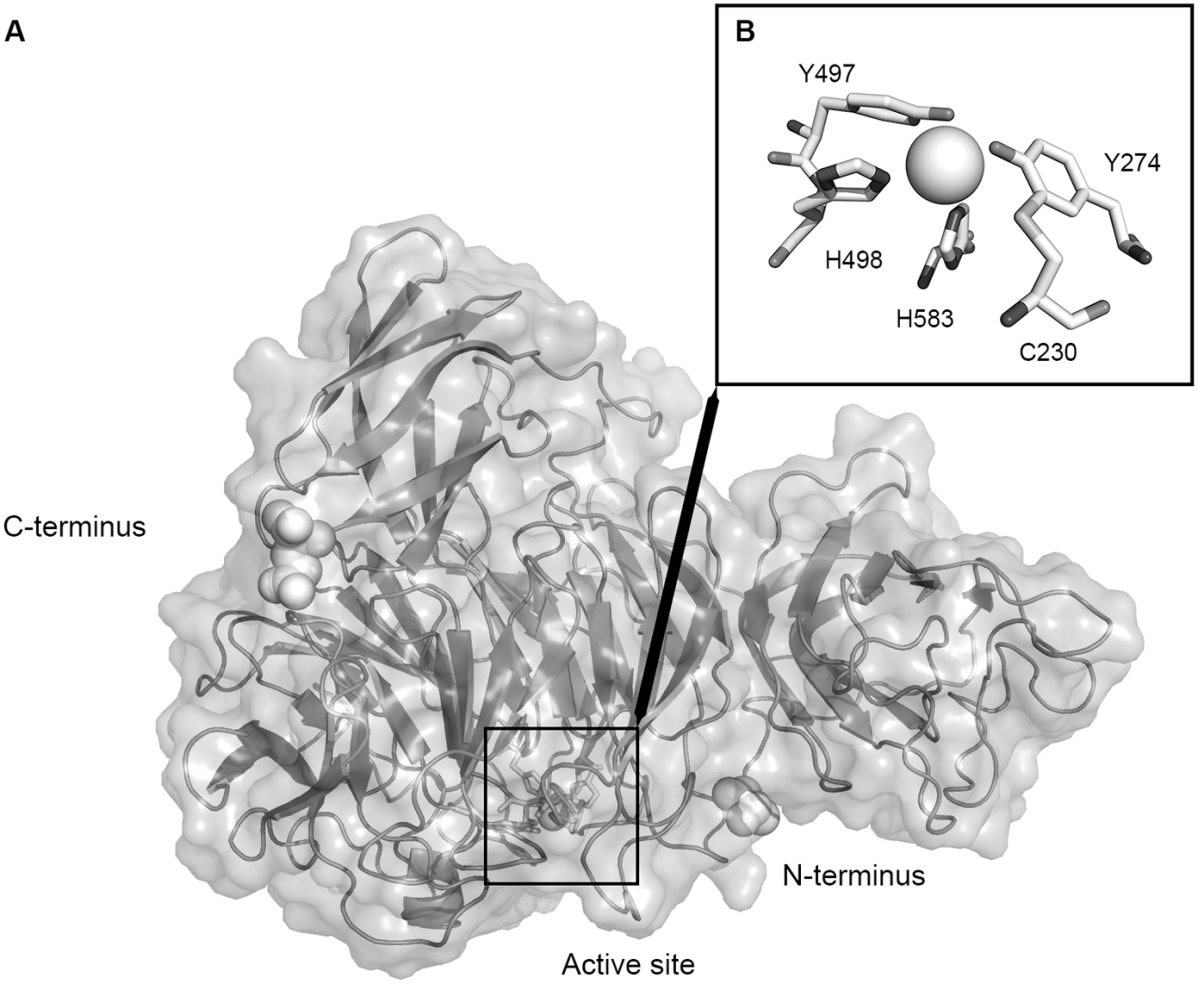
3D Structure of GalOx of *F. oxysporum*. A: Overall structure showing the predominantly β-structure. The N-terminus, C-terminus and the copper atom in the active site are highlighted. B: The active site of GalOx showing the copper ligands and the thioether cross-link. The structural model was generated by homology modelling based on the published structure of mature GalOx from *F. graminearum* (PDB 1gog) using SWISS_MODEL.

**Figure 3:**
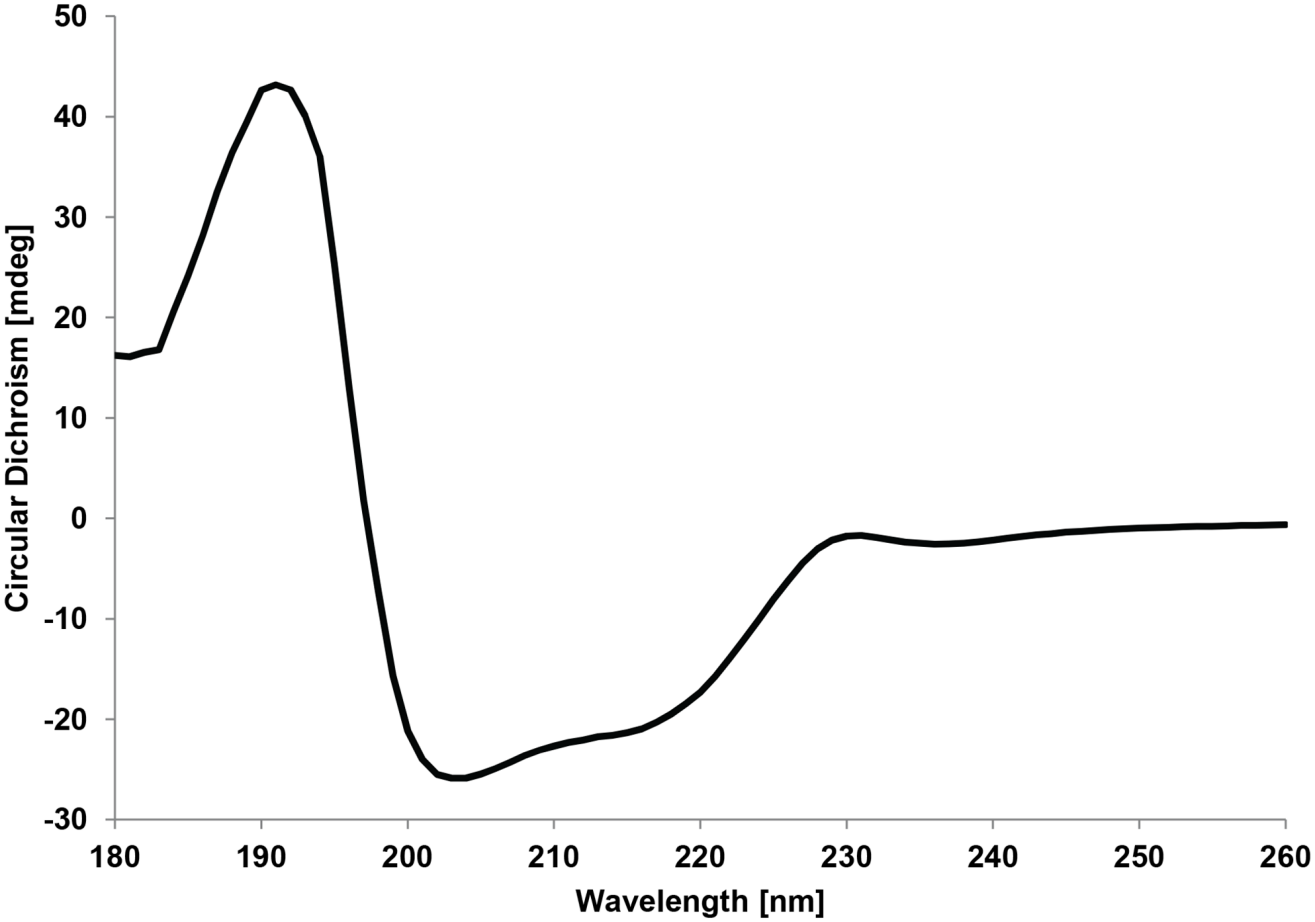
Far UV circular dichroism spectrum of GalOx from *F. oxysporum*.

The amino acid sequence derived from the *F. oxysporum gao* gene was used to generate a sequence alignment with the published sequence of *F. graminearum* using Clustal Omega [34] (Figure 1). A three-dimensional homology model was created based on the published structure of mature GalOx (1gog) from *F. graminearum* [3] using SWISS-MODEL [35]–[37] (Figure 2). Despite of a similarity of only 82% the amino acids directly in the active site (Figure 2B) as well as the second shell around it are completely conserved. The amino acid residues responsible for copper binding are also entirely conserved as are the amino acids in the substrate-binding pocket. To test the correctness of the predicted structure far infrared CD spectra were recorded for GalOx from *F. oxysporum* (Figure 3) and compared with CD spectra from *F. graminearum* (data not shown) [30]. Both spectra had maxima at 190 and 230 nm, a minimum at 203 nm, and are essential identical to the one published for *F. graminearum* [38].

Expression of the *gao* gene in *E. coli* and *P. pastoris* was compared to find a suitable host for the production of recombinant GalOx. For expression in *E. coli* suitable restriction sites were introduced, and the full-length gene including the prepro sequence was cloned into the vector pET21a, which adds a C-terminal His-tag. After transformation of the plasmid into *E. coli* BL21 (DE3) the expression host was cultivated in double-concentrated LB medium and 5% lactose was used as inducer for expression of the *gao* gene. Routinely 5.7 mg L^−1^ of active, soluble GalOx (as calculated from the volumetric activity of 380 U L^−1^ and a specific activity of the homogenous enzyme of 65.4 U mg^−1^) were obtained in shaking flask cultivations after incubation at 25°C for 16 h. This translates to a space-time yield of 0.36 mg L^−1 ^h^−1^, which is tenfold higher than the yield reported for native GalOx from *F. graminearum* expressed in *E. coli* [18], [22]. It can be expected that the use of an optimized fermentation protocol can substantially improve upon these yields [30]. No soluble, active protein could be obtained by expression of the *gao* gene without its prepro sequence in *E. coli* (data not shown).

For expression in *P. pastoris* two different strategies were used. To test whether *P. pastoris* is able to recognize the secretion signal of *F. oxysporum* the full-length *gao* cDNA (including the native prepro signal sequence) was cloned into the vector pPIZB, adding again a C-terminal His-tag. After 8 days of growth in BMMY medium with methanol feed the activity in the supernatant reached a maximum volumetric activity of 700 U L^−1^, corresponding to 10.6 mg of recombinant enzyme per L medium. This translates to a space-time yield of 0.06 mg L^−1 ^h^−1^, which is significantly lower than the yield obtained in *E. coli*. No detectable GalOx activity was found in the biomass after cell disruption. In a second approach the native signal sequence of GalOx was replaced with that of the α-factor of *S. cerevisiae* in the pPICZαA vector. In this case a volumetric activity of 200 U L^−1^ (corresponding to approx. 3 mg L^−1^ recombinant GalOx and a space-time yield of 0.014 mg L^−1 ^h^−1^) was found in the supernatant after 8 days of cultivation. Again no GalOx activity was detectable in the biomass. *P. pastoris* is therefore not only able to recognize the GalOx signal peptide but the production of active enzyme is also >3-fold increased compared to when the α-factor signal sequence is used. This is in contrast to a previous report on the expression of recombinant GalOx from *F. graminearum* in *P. pastoris* [25], where expression of the full-length native sequence resulted in a mixture of partly processed and mature galactose oxidase, while the use of the glucoamylase signal peptide resulted in higher expression yields. In a report comparing the expression of GalOx from *F. graminearum* in different host a threefold increase in volumetric productivity was found for expression in *P. pastoris* compared to *E. coli* [18].

### Purification and prove of the thioether bond in GalOx

Based on the His-tag, which is provided by all three expression vectors, the protein could be conveniently purified by IMAC followed by a polishing step of size-exclusion chromatography. The His-tag was added C-terminally since this will position the tag away from the active site of the enzyme (Figure 2A) in contrast to an N-terminal positioning. An interference of the tag with the active site is therefore not likely.

**Figure 4:**
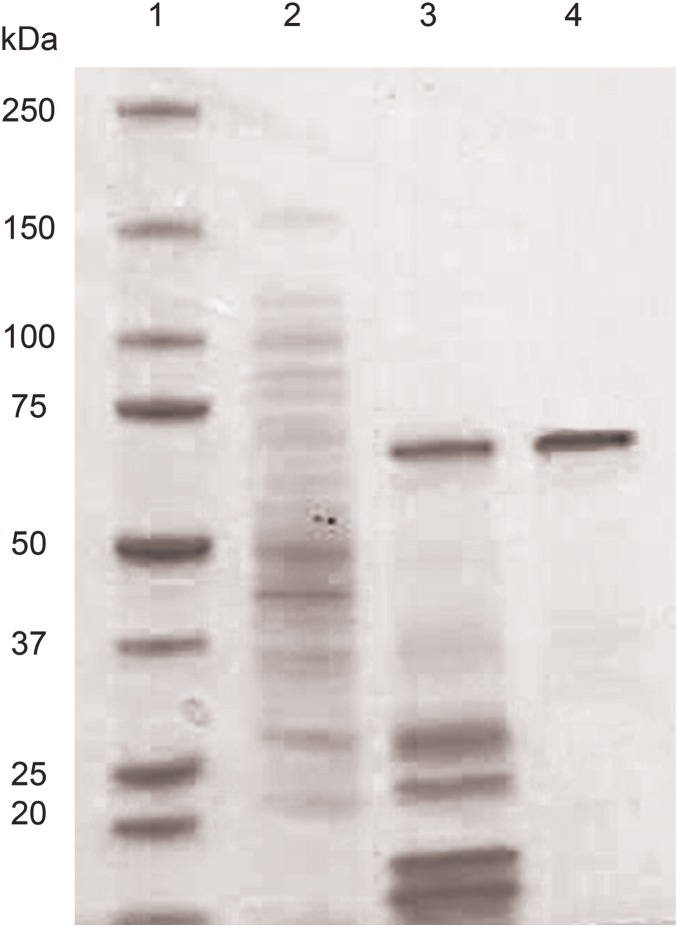
Electrophoresis analysis of crude extract and purified GalOx expressed in *E. coli*. Lane 1, Precision Plus Protein Standard (BioRad); lane 2, GalOx crude extract; lane 3, GalOx after IMAC; lane 4, GalOx after size-exclusion chromatography.

A typical purification procedure of GalOx expressed in *E. coli* is summarized in Table 1. The enzyme was purified 28-fold from the crude cell extract with a yield of 22% and a specific activity of 65.4 U mg^−1^ was obtained for the purified enzyme. The two-step purification procedure yielded an apparently homogenous protein, as judged by SDS-PAGE (Figure 4). Full-length GalOx (including the native prepro signal sequence) expressed in *P. pastoris* was purified six-fold with a yield of 33% and a specific activity of 63.2 U mg^−1^. GalOx expressed in *P. pastoris* using the α-factor signal of *S. cerevisiae* was purified 22-fold with a yield of 19% and a specific activity of 61. U mg^−1^ (results not shown).

**Table 1:** Purification of recombinant GalOx expressed in *E. coli.*

	Protein(mg)	Totalactivity (U)	Specific activity(U/mg)	Purification(fold)	Yield (%)
Crude extract	293	676	2.31	1	100
Affinity chromatography	10.9	525	48.2	21	78
Size-exclusion chromatography	2.32	152	65.4	28	22

A protein band of 72 kDa was observed by SDS-PAGE analysis of GalOx expressed in *E. coli* rather than a 74-kDa band predicted by the DNA sequence including the prepro signal sequence and the His-tag (Figure 4). The faster migration rate indicates that the thioether bond between Cys230 and Tyr274 is formed in the enzyme [14], [28]. Similar results are found for GalOx expressed in *P. pastoris* with native prepro sequence and α-factor, respectively. Both migrated on SDS-PAGE with an apparent molecular weight of 70 kDa rather than 72 kDa calculated from the DNA sequence after processing of the signal sequence.

Previous work [3], [39] has demonstrated that GalOx contains a covalent crosslink between a tyrosine and a cysteine residue, which is required for activity of the enzyme and serves as a protein cofactor together with the mononuclear copper center in GalOx. In the original fungal host this crosslink results from a self-processing reaction during protein expression [4], [14]. In previous publications the formation of this thioether bond was proven by high-resolution crystallography [3] or indirect methods such as modified migration on SDS-PAGE and spectroscopic approaches [14], [15], [28], [39]. In this work we wanted to confirm the formation of the thioether bond during heterologous expression of GalOx in *E. coli* and *P. pastoris* by a direct method using mass spectrometry. For this purpose an in-gel digestion of the purified protein was performed with different protease chemistry and the obtained peptide mixtures were analyzed using MALDI mass spectrometry.

**Figure 5:**
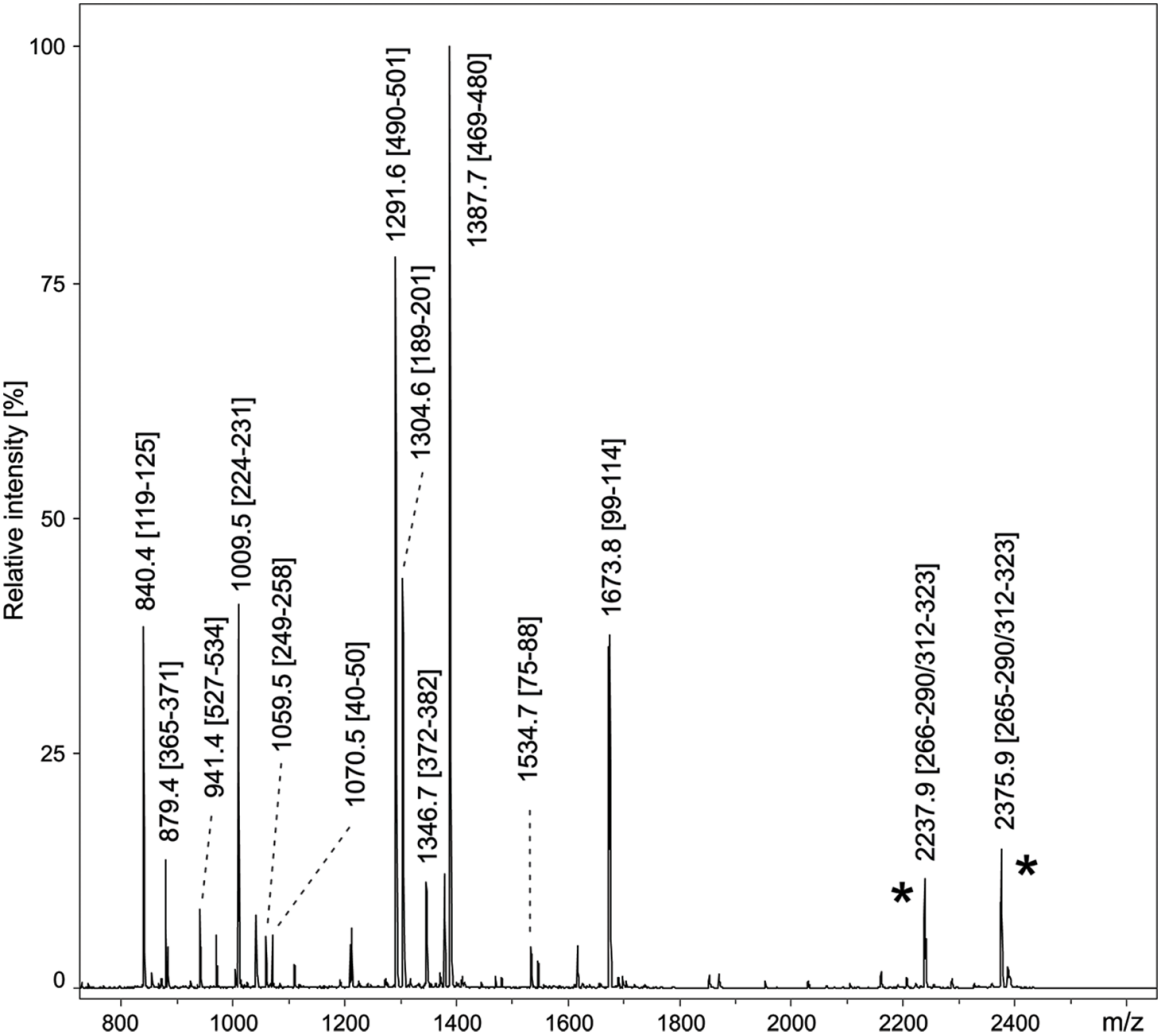
MALDI-TOF peptide mass map of GalOx expressed in *E. coli*. The peptides were generated by sequential digestion using trypsin and Asp-N. The peak labels correspond to the [M+H]^+^ ions of the obtained peptide fragments and their positions in the GalOx sequence. The spectrum also shows two intense signals (marked with asterisk) at m/z 2237.9 and 2374.9 related to the cross-linked peptides 266–274/312–323 and 265–274/312–323, respectively. The identity of the cross-linked peptides was verified by MS/MS fragmentation.

After digestion with trypsin a tiny peak at 3914.7 m/z corresponding to the cross-linked bispeptide 265–290/312–323 was observed. No analogous peak was found in the mass spectrum of the Asp-N digest, presumably because of the rather high molecular mass of the expected cross-linked bispeptide with the protonated mass of 5066.4. To reduce the size of the cross-linked peptides and thus increase their ionization efficiency, different sequential digestions using trypsin and Asp-N were performed. When digesting first with Asp-N and subsequently with trypsin an intense peak of the shorter cross-linked peptide 266–274/312–323 was obtained, whereas the two cross-linked peptides 265–274/312–323 (2374.9 m/z) as well as 266–274/312–323 (2237.9 m/z) were observed when a trypsin/Asp-N digestion was applied (Figure 5). The identity of the cross-linked peptides was confirmed by MS/MS fragmentation on a MALDI-TOF instrument and also using high accuracy measurements on a FT-ICR mass spectrometer. A detailed list of the detected cross-linked peptides with their corresponding theoretical molecular weight can be found in Table 2. These data unequivocally confirm that the thioether bond is indeed correctly formed during the expression of GalOx from *F. oxysporum* both in *E. coli* and *P. pastoris*. There were no signals in MS indicating the presence of the corresponding peptides without the thioether crosslink. To the best of our knowledge mass spectrometry has not yet been used to characterize the formation of the Tyr-Cys crosslink in GalOx. Mass spectrometry was used to identify this crosslink in the orphan metalloprotein BF4112, however, the cross-linked bispeptides obtained after proteolytic digestion had to be separated by HPLC since the signal was too weak to be identified in the mixture of peptides [40]. This problem was not encountered with the GalOx digests and a strong signal was obtained.

**Table 2:** MALDI-FTMS data of the cross-linked peptides produced by in-gel digestion of GalOx. The cross-linked amino acids C230 and Y274 are highlighted.

Protein	Digestion conditions	Sequence of cross-linked peptide	[M+H]^+^theor.	[M+H]^+^exper.	Mass error[ppm]
***GalOx***	trypsin	HDMF**C**PGISMDGNGQVVVTGGNDAQK/G**Y**QSSATLSNGR	3914.7489	3914.7386	2.6
***E. coli***	trypsin/AspN	DMF**C**PGISM/G**Y**QSSATLSNGR	2237.9623	2237.9587	1.6
	trypsin/AspN	HDMF**C**PGISM/G**Y**QSSATLSNGR	2375.0212	2375.0172	1.7
	AspN/trypsin	DMF**C**PGISM/G**Y**QSSATLSNGR	2237.9623	2237.9637	0.6
***GalOx***	trypsin	HDMF**C**PGISMDGNGQVVVTGGNDAQK/G**Y**QSSATLSNGR	3914.7489	3914.7390	2.5
***P. pastoris***	trypsin/AspN	DMF**C**PGISM/G**Y**QSSATLSNGR	2237.9623	2237.9603	0.9
	trypsin/AspN	HDMF**C**PGISM/G**Y**QSSATLSNGR	2375.0212	2375.0166	1.9
	AspN/trypsin	DMF**C**PGISM/G**Y**QSSATLSNGR	2237.9623	2237.9609	6.6

### Biochemical characterization

**Figure 6:**
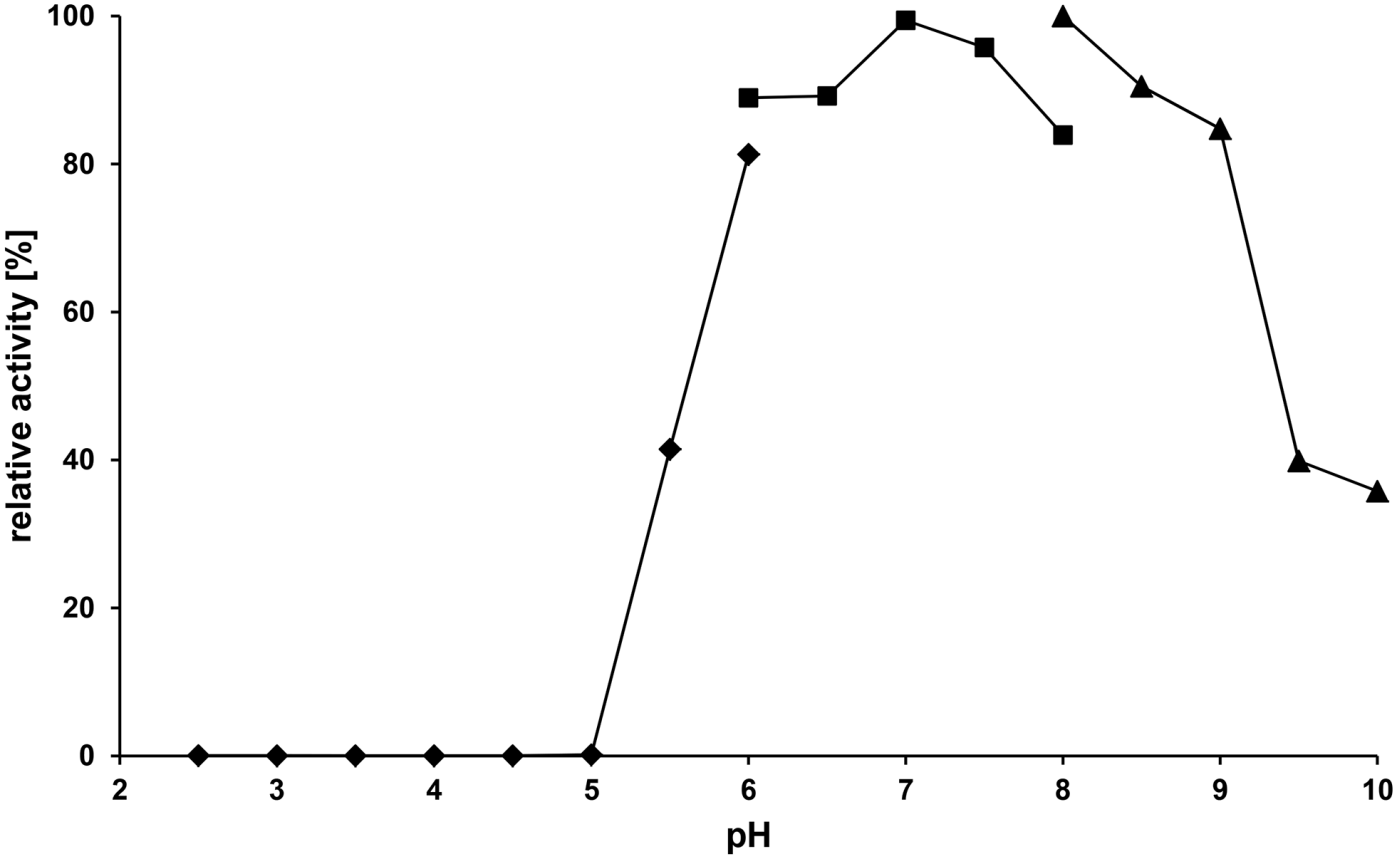
Effect of the pH on the activity of GalOx expressed in *E. coli*. The buffers used were 50(♦), 50 mM phosphate (_▀_) and 50 mM Tris (▴).

All data on the biochemical characterization were obtained with GalOx from *F. oxysporum* expressed and purified from the cell extract of *E. coli*. GalOx exhibited a broad pH optimum (Figure 6), with more than 80% of the maximum activity retained between pH 6.0 and 9.0. Maximum activity occurred at pH 7.0 (phosphate buffer) and 8.0 (Tris buffer), respectively. At pH values below 5.0 the enzyme lost its activity. The optimum pH of recombinant GalOx from *F. oxysporum* described here is in good agreement with data reported previously for the native enzyme purified from other sources [5], [7], [41].

**Figure 7:**
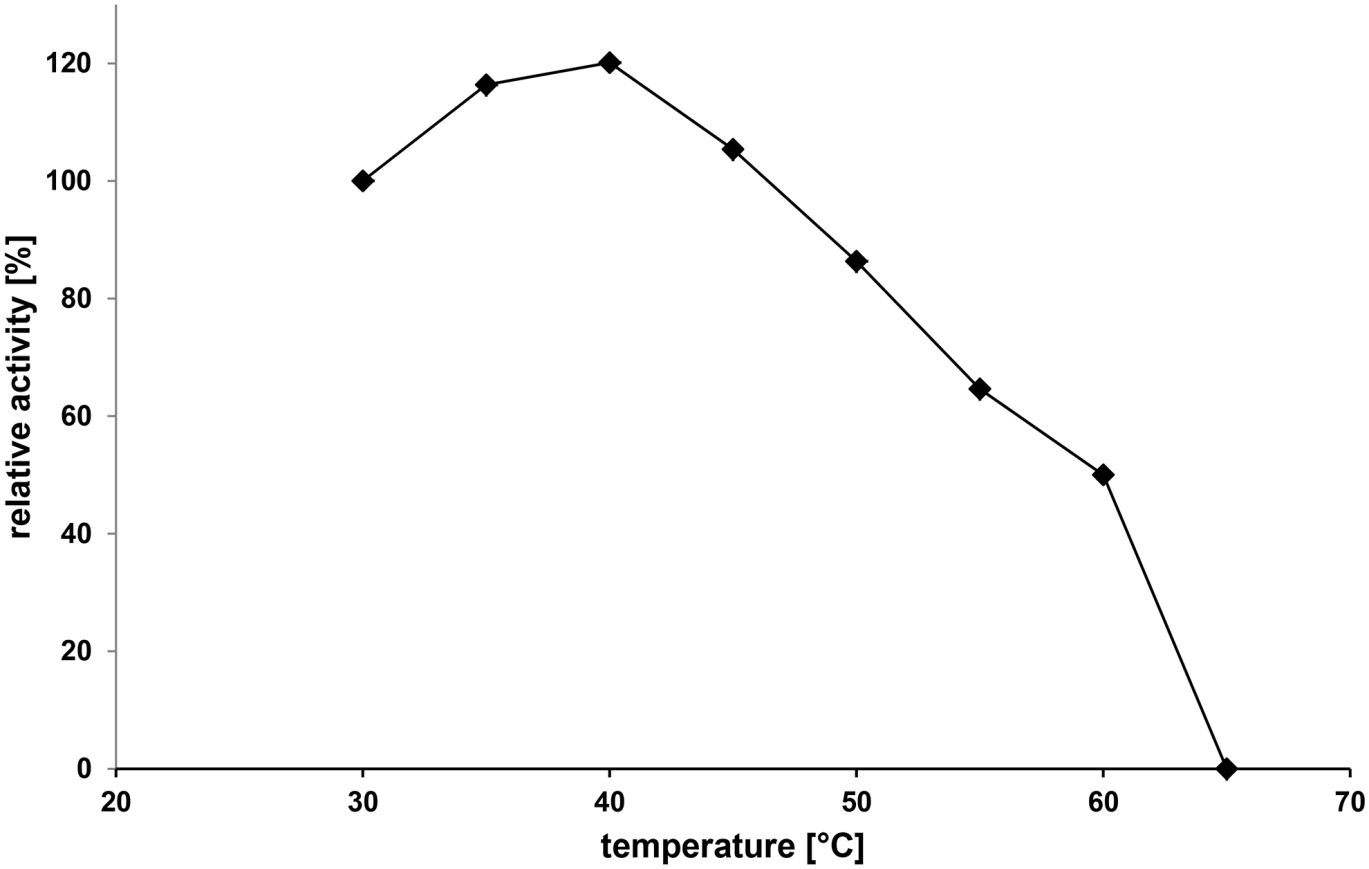
Temperature dependence of the activity of GalOx expressed in *E. coli.*

**Figure 8:**
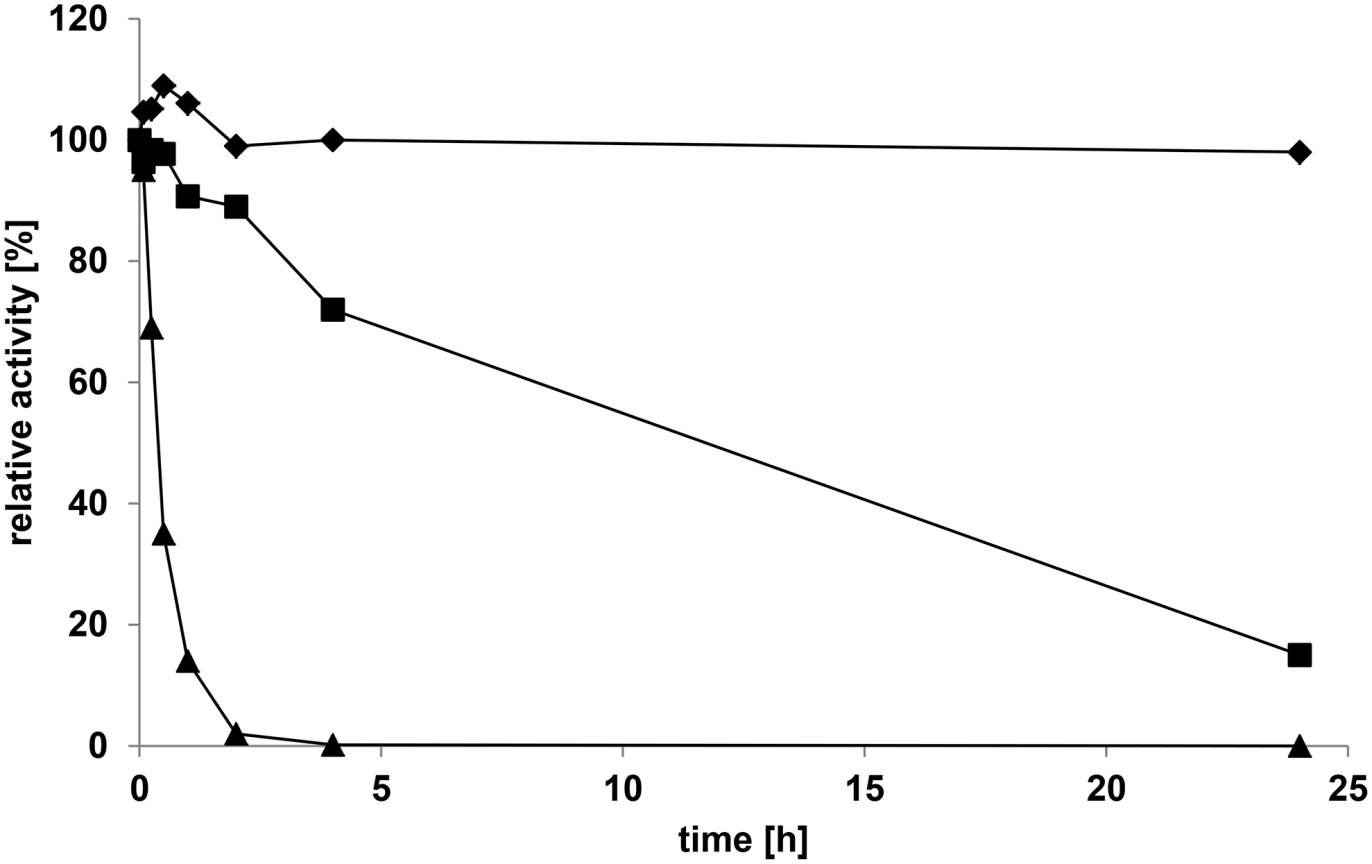
Thermal stability of GalOx from *F. oxysporum* produced in *E. coli*. Preincubation of GalOx in 20°C (♦), 40°C (_▀_) and 50°C (▴), respectively, for various time points.

Figure 7 shows the effect of temperature on GalOx activity at pH 7.0. The enzyme was active from 30 to 60°C with an optimal temperature of 40°C for the 3-min assay. Studies of thermal stability of GalOx (Figure 8) showed that the enzyme is stable at 30°C for at least 24 h. The calculated half-life was 8.77 h, 52 min, and 0.5 min for incubation at 40°C, 50°C, and 60°C, respectively. The *T_50_* values (the temperature at which the enzyme loses 50% of its initial activity following incubation for 10 min) of GalOx calculated from these data was 52°C. Additionally, calorimetric studies of the thermal denaturation of GalOx were performed. Using phosphate buffer at pH 7 the DSC gave a single endothermic peak with a *Tm* of 64.6°C. To test if different buffer systems have an influence on the temperature stability of the enzyme DSC measurements were performed with citric acid and Tris buffer, each at pH 7. A *Tm* of 63.7°C and 63.9°C was measured for citric acid and Tris buffer, respectively. Hence these buffer systems have only a small effect on the stability of the enzyme. Based on our studies, the thermostability of GalOx from *F. oxysporum* expressed in *E. coli* is significantly lower than the thermostability of the enzyme from other *Fusarium* strains [7], [22].

GalOx has a broad substrate specificity, which is one of the most important characteristics of the enzyme [12]. Steady-state kinetic constants for different substrates were determined using oxygen (air) as electron acceptor. The initial rates of substrate turnover were recorded using different substrate concentrations in the standard ABTS assay at 30°C and pH 7.0. Kinetic data are summarized in Table 3. The highest catalytic efficiency (*k*_cat_/K_m_) was found for 1-methyl-β-D-galactopyranoside (2.2 mM^−1 ^s^−1^) followed by D-galactose (2.0 mM^−1 ^s^−1^). Both substrates give similar K_m_ values (45 mM and 47 mM, respectively). The K_m_ value for D-galactose of recombinant GalOx from *F. oxysporum* is comparable to that of recombinant GalOx from *F. graminearum* (35 mM) [30], both produced in *E. coli*. These values are lower than the data for native GalOx from *F. graminearum* with 67 mM [28]. GalOx has a higher affinity to raffinose (lower K_m_) than to D-galactose but due to an increased *k*_cat_ value the catalytic efficiency is lower for raffinose. Previous studies [42] obtained very similar Michaelis constants for raffinose and melibiose by the native GalOx from *P. circinatus* (25 mM and 45 mM, respectively) as well as the recombinant GalOx from *F. oxysporum* (23 mM and 41 mM, respectively) although the K_m_ value for D-galactose is five-times higher for *P. circinatus* (240 mM). The activity of the enzyme with raffinose, lactose, melibiose, 1-methyl-β-D-galactopyranoside, 1-methyl-α-D-galactopyranoside, lactitol and lactulose indicates that GalOx can oxidize galactose derivatives with substitutes at the carbon-1 site, as previously reported by Alberton *et al.* [7]. GalOx is also not sensitive to changes at the C2 group according to the results with 2-deoxy-D-galactose as substrate. This conclusion stands in accordance with previous studies [43] about the reactivity of recombinant GalOx from *F. graminearum* produced by a fungal host against D-talose and D-galactosamine, respectively. Because of the high sensitivity of GalOx to the stereo configuration of the C4 hydroxyl group D-glucose is not a substrate for the enzyme. The here reported substrate specificity is in good agreement with previously published results [12], [43], [44], which is not surprising considering the highly conserved active site environment of the different galactose oxidases. Furthermore, it indicates that the C-terminal His-tag added to recombinant GalOx from *F. oxysporum* does not interfere with the catalytic activity. The relatively high K_m_ values for different substrates for GalOx seem to be a consequence of the broad substrate specificity of the enzyme resulting in an active site capable of binding a range of different substrates, but therefore being relatively weak at binding any particular substrate [45].

**Table 3:** Apparent kinetic constants of GalOx produced in *E. coli* for several electron donor substrates.

Substrate	*V*_max_ (µmol min^−1 ^mg^−1^)	Relativeactivity (%)	K_m_ (mM)	*k*_cat_ (s^−1^)	*k*_cat_/K_m_ (mM^−1 ^s^−1^)
D-galactose	78	100	47	95	2.0
1-methyl-β-D-galactopyranoside	81	104	45	99	2.2
raffinose	23	29	23	28	1.2
lactose	43	55	685	52	0.076
D-melibiose	62	79	41	75	1.8
1-methyl-α-D-galactopyranoside	47	60	41	57	1.4
lactulose	63	81	102	77	0.75
2-deoxy-D-galactose	39	50	47	47	1.0
lactitol	55	71	470	67	0.14

## Conclusions

GalOx is of interest for various biotechnological applications, ranging from biosensors to diagnostic use in medicine as well as biocatalysis [12]. Because of this interest, more detailed knowledge about GalOx from other sources is of interest. The *gao* gene coding for GalOx from *F. oxysporum* can be easily expressed in the preeminent microbial expression hosts *E. coli* and *P. pastoris* even without codon optimization or further amino acid substitutions that have been reported to improve expression in *E. coli* [18], [22]. GalOx from *F. oxysporum* is very well comparable in its biochemical and catalytic properties to other fungal GalOx, which is not surprising when considering the well-conserved geometry of the active site and the substrate-binding site in these different enzymes.

Mass spectrometry as a tool for the detection of the Tyr-Cys crosslink could find wider application in the characterization of this unique protein cofactor in GalOx but also in related enzymes. Mass spectrometry does not provide quantitative data for these crosslinks, but is a rapid and standard methodology widely established by now, and thus could replace the indirect methods that are commonly used to characterize unequivocally the formation of the unique protein cofactor in GalOx as well as in related enzymes.
